# Preparation of Green Anti-*Staphylococcus aureus* Inclusion Complexes Containing Hinoki Essential Oil

**DOI:** 10.3390/foods12163104

**Published:** 2023-08-18

**Authors:** Peifu Kong, Kotchaporn Thangunpai, Ainun Zulfikar, Shunsuke Masuo, Junichi Peter Abe, Toshiharu Enomae

**Affiliations:** 1 Degree Programs in Life and Earth Sciences, University of Tsukuba, Tsukuba 305-8572, Ibaraki, Japan; peifukong@163.com (P.K.); kotchapornjee@gmail.com (K.T.); ainun@lecturer.itk.ac.id (A.Z.); 2 Materials and Metalurgical Engineering Department, Institut Teknologi Kalimantan, Balikpapan 76127, Indonesia; 3 Faculty of Life and Environmental Sciences, University of Tsukuba, Tsukuba 305-8572, Ibaraki, Japan; masuo.shunsuke.fp@u.tsukuba.ac.jp (S.M.); abe.junichi.p.gn@u.tsukuba.ac.jp (J.P.A.)

**Keywords:** anti-*Staphylococcus aureus*, β-cyclodextrin/2-hydroxypropyl-β-cyclodextrin, hinoki essential oil, inclusion complexes, ultrasound-assisted kneading

## Abstract

This study aimed to prepare anti-*Staphylococcus aureus* inclusion complexes (ICs) of Hinoki essential oil (HEO) with β-cyclodextrin (β-CD) and 2-hydroxypropyl-β-cyclodextrin (2-HP-β-CD). An ultrasound-assisted kneading method was applied for the complexation for the first time. The recovery yield, embedding fraction and loading capacity of the HEO/β-CD ICs were 92.5%, 78.0% and 11.9%, respectively, while the corresponding values were 80.8%, 73.7% and 12.9% for the HEO/2-HP-β-CD ICs. As well, a comparative study confirmed the efficiency of the ultrasound-assisted kneading method was higher than the traditional kneading method. The results of SEM, XRD, GC-MS and FT-IR suggested the successful formation of ICs. A significant anti-*Staphylococcus aureus* activity of the fabricated ICs was demonstrated using a colony counting method. Notably, when the dose in liquid culture medium was 20 g L^−1^, inhibitory rates of 99.8% for HEO/β-CD ICs and 100% for HEO/2-HP-β-CD ICs were achieved. Furthermore, the hydrophilic property of the ICs was proved by water contact angle measurements, implying they have the potential to act as anti-*Staphylococcus aureus* agents for blending with hydrophilic biodegradable materials for diverse food packaging utilizations.

## 1. Introduction

Staphylococcal food poisoning and diseases caused by *Staphylococcus aureus* (*S. aureus*) have grown into serious public health concerns across the world [1,2]. *S. aureus* is well known for its infection in the integumentary system and underlying soft tissues. Indeed, this pathogen can target a wide range of organ systems within the human body, resulting in severe repercussions after the ingestion of contaminated food items. As a result, enhancing the avoidance of *S. aureus* transmission in food has piqued the interest of researchers. From a packaging standpoint, antibacterial food packaging has emerged as a promising and efficient approach. To date, a variety of active substances such as nanoparticles of silver, gold and zinc oxide have been successfully incorporated into antibacterial packaging [3,4,5,6]. Nevertheless, the use of these substances in food packaging has caused widespread concern among consumers due to their potential toxicity [7]. Therefore, it is extremely favorable to seek out green antibacterial agents.

Substances obtained from botanical sources are satisfactory alternative options. Essential oils (EOs) are secondary metabolites that can be extracted from various plant portions. They consist of a complex mixture of hydrophobic bioactive molecules that give plants their characteristic odor. EOs that are generally recognized as safe (GRAS) additives have been frequently used as flavoring agents in foods, medicine and cosmetics [7,8]. Hinoki (*Chamaecyparis obtusa*) is a coniferous tree that has been utilized for construction and furniture for centuries in East Asia. The Hinoki essential oil (HEO) has been commercially used as a functional fragrance in detergent, toothpaste and air purifier [9,10]. Especially, it has been validated as effective for regulating brain function and autonomic nervous system activity, particularly in promoting a state of calmness and facilitating decompression [10,11]. Several studies on the chemical compositions of HEO have demonstrated that it predominantly comprises terpenes (e.g., pinene, limonene and cadinene) and terpenoids (e.g., terpinene-4-ol and cadinol). These molecules have been discovered to be anti-oxidant, insecticidal and antimicrobial [12]. However, to the best of our knowledge, the application of HEO in food or food packaging has not yet been reported. In the study of Park et al. [13], HEO exhibited an inhibitory effect on the growth of *S. aureus,* and this finding suggests that the HEO has the potential to be utilized as an anti-*S. aureus* agent for food packaging. Nonetheless, HEO is extremely volatile and susceptible to oxidative damage when exposed to light, oxygen and heat [7]. Furthermore, the property of its high hydrophobicity severely hinders the adaptation to some biodegradable food packaging materials like chitosan, cellulose and alginate [14,15].

Complexation using β-cyclodextrin (β-CD) and 2-hydroxypropyl-β-cyclodextrin (2-HP-β-CD) is a prospective technology for avoiding the aforementioned drawbacks [16,17]. β-CD is a cyclic oligosaccharide comprising seven D-glucopyranose units linked together by α-1,4-glucose bonds and these units form a hollow truncated-cone-shaped structure. This special structure endows it with a hydrophobic cavity and a hydrophilic surface which can form host–guest systems called inclusion complexes (ICs) with a wide variety of hydrophobic molecules such as EOs [18,19]. 2-HP-β-CD is a derivative of β-CD by substituting hydrogen atoms of hydroxyl groups with hydroxypropyl groups, resulting in a much higher solubility and a greater complexation ability reported by Gharibzahedi et al. [20]. Moreover, they are classified as nontoxic biodegradable products and GRAS, allowing them to be used in food applications [21]. There are numerous approaches for the formation of ICs including kneading, co-precipitation, freeze drying, spray drying and so forth. Among them, the kneading method is frequently applied and regarded as a convenient technique for EOs since it does not require heat or a complicated procedure [22]. However, its low complexation efficiency or long process time increases the economic and time costs for subsequent large-scale production. Hădărugă et al. [23] performed a 30 min of kneading process for the complexation of Danube common nase oil with β-CD and 2-HP-β-CD and reported a recovery yield of merely 77.7% and 79.9%. In a recent study reported by Adjali et al. [24], clove essential oil was complexed with 2-HP-β-CD via the kneading method for 50 min, but the embedding fraction of clove essential oil was as low as 50%.

Ultrasound as a non-thermal technology also has the potential for molecule complexation due to its simplicity, high energy and great efficiency [16,25]. Recent research has highlighted the superior performance of ultrasound in the ICs preparation. For example, Siva et al. [26] prepared the ICs of isoeugenol and cuminaldehyde with methyl-β-CD using an ultrasound process to improve the aqueous solubility and thermal stability of molecules. Conceivably, a combination of the kneading method with ultrasound may increase complexation efficiency while reducing the total preparation time. To the best of our knowledge, there is no report in the literature regarding this, and hence the aim of this study was to complex HEO with β-CD and 2-HP-β-CD using an ultrasound-assisted kneading method to prepare anti-*S. aureus* ICs. Subsequently, the complexation efficiency of the ultrasound-assisted kneading method was well compared to the traditional kneading method. The obtained ICs were characterized by scanning electron microscopy (SEM), X-ray diffractometry (XRD), gas chromatography-mass spectrometry (GC-MS), Fourier transform infrared spectroscopy (FT-IR) and contact angle meter. In addition, the anti-*S. aureus* activity was evaluated via a colony counting method.

## 2. Materials and Methods

### 2.1. Materials

β-CD (Wako 1st Grade) and 2-HP-β-CD were obtained from FUJIFILM Wako Chemicals (Osaka, Japan) and Tokyo chemical industry Co., Ltd. (Tokyo, Japan), respectively. HEO, extracted from the xylem of Hinoki, was bought from INSCENT CO. (Nagano, Japan). Ethanol (guaranteed reagent, 99.5%) was purchased from FUJIFILM Wako Chemicals. All other chemicals and solvents were analytical grade and were used directly without further purification.

*S. aureus*, derived from ATCC 25923, was bought from Microbiologics, Inc. (Saint Cloud, MN, USA). Tryptic soy agar (TSA) and Tryptic soy broth (TSB) were purchased from Geno Technology Inc. (St. Louis, MO, USA).

### 2.2. Preparation of ICs

The ICs prepared with β-CD and 2-HP-β-CD using the ultrasound-assisted kneading method were denoted as “HEO/β-CD ICs” and “HEO/2-HP-β-CD ICs”, respectively. The mass ratio of the host (β-CD or 2-HP-β-CD) to guest (HEO) was controlled as 6:1 in this study based on the result reported by Bai et al. [27]. For HEO/β-CD ICs, firstly, a total of 2 g β-CD, 6 mL 40% ethanol, and 0.33 g HEO were put in a ceramic mortar, followed by a constant manual kneading for 5 min. Then, the mortar was transferred into an ultrasonic water bath (MCD-27, AS ONE Corporation, Osaka, Japan). The liquid level of the ultrasonic water bath was adjusted at a position slightly lower than the highest point of the mortar, and the mortar was treated at 40 kHz and 23 °C (± 1 °C) for 5 min. Subsequently, the mortar was taken out of the ultrasonic water bath and another 5 min of manual kneading was performed. The resulting product was dried in a desiccator with sufficient silica gels at 23 °C (± 1 °C) for 48 h. Afterwards, the dried product was rinsed with ethanol twice via a vacuum filtration process to remove the excess HEO on the surface, followed by drying in the desiccator for another 24 h. Finally, the remaining product (supposed to be HEO/β-CD ICs) was stored in a hermetically sealed brown glass bottle at 23 °C (±1 °C) for subsequent characterization. For HEO/2-HP-β-CD ICs, the only distinction in the preparation procedure compared to the HEO/β-CD ICs was that the amount of 40% ethanol added was 2 mL instead of 6 mL with a consideration of a much higher solubility of 2-HP-β-CD in 40% ethanol than β-CD. In addition, the blank samples were prepared without any addition of HEO in the same condition as described above and referred to as “β-CD blank” and “2-HP-β-CD blank”, respectively. For comparison to the traditional kneading method, ICs were also prepared by simply kneading for 15 min, and referred to as “HEO/β-CD ICs control” and “HEO/2-HP-β-CD ICs control”, respectively.

### 2.3. Characterization of ICs

#### 2.3.1. Complexation Efficiency

Complexation efficiency was evaluated based on the three factors: recovery yield (RY), embedding fraction (EF), and loading capacity (LC) calculated using Equations (1)–(3) as follows.

(1) $$\mathrm{RY}\;(\%)=\frac{\;\mathrm{weight}\;\mathrm{of}\;\mathrm{recovered}\;\mathrm{ICs}\;(g)}{\mathrm{initial}\;\mathrm{host}\;\mathrm{material}\;\mathrm{weight}\;(g)+\mathrm{initial}\;\mathrm{HEO}\;\mathrm{weight}\;(g)}\times \;100,$$

(2) $$\mathrm{EF}\;(\%)=\frac{\mathrm{weight}\;\mathrm{of}\;\mathrm{embedded}\;\mathrm{HEO}\;(g)}{\mathrm{initial}\;\mathrm{HEO}\;\mathrm{weight}\;(g)}\;\times \;100$$

(3) $$\mathrm{LC}\;(\%)=\frac{\mathrm{weight}\;\mathrm{of}\;\mathrm{embedded}\;\mathrm{HEO}\;(g)}{\mathrm{weight}\;\mathrm{of}\;\mathrm{recovered}\;\mathrm{ICs}\;(g)}\;\times \;100$$

The weight of embedded HEO was determined as follows: a calibration curve showing the correlation of the HEO concentration and its absorbance at a wavelength of 240 nm was previously constructed using an ultraviolet-visible spectrophotometer (UV-3100 PC, Shimadzu Corporation, Kyoto, Japan) according to the methodology described in our former study [28]. A total of 0.04 g ICs were extracted with 40 mL ethanol via a vortex mixer (HS120214, Heathrow Scientific, LLC, Vernon Hills, IL, USA) at 3000 r min^−1^ for 10 min, followed by centrifugation at 1700× *g* for 10 min. Then, the supernatant was filtered and its absorbance at 240 nm was measured. Finally, the weight of HEO embedded in the ICs was calculated using the calibration curve.

#### 2.3.2. Morphological Observation

The morphological structures of the prepared ICs were examined using an SEM (TM4000Plus, Hitachi, Ltd., Tokyo, Japan) in a backscattered electron mode at an accelerated voltage of 15 kV. This observation was conducted in a low-vacuum situation to minimize the evaporation of molecules embedded in the ICs.

#### 2.3.3. GC-MS Analysis

The confirmation of the formation of ICs was achieved via a comparative analysis of the volatile molecules embedded in ICs and those of HEO using a GC-MS. The extraction of volatile molecules embedded in the ICs was conducted as follows: a total of 0.2 g ICs was subjected to extraction using 40 mL ethanol via the vortex mixer at 3000 rpm for 10 min and centrifugated at 1700× *g* for 10 min. Subsequently, 1 mL supernatant was filtered and diluted to a final volume of 15 mL using ethanol. Finally, 2 µL of the resulting solution (named “ethanol extract of HEO/β-CD ICs” and “ethanol extract of HEO/2-HP-β-CD ICs”) was introduced into a GC-MS-QP2010Plus (Shimadzu Corporation, Kyoto, Japan) equipped with an AOC-30/20i+s autosampler. The separation was achieved using an Rtx-5MS column with dimensions of 30 m × 0.25 µm and a film thickness of 0.25 µm. For the HEO sample, it was diluted 3 × 10^4^ times with ethanol before injection to GC-MS for the detection of volatile molecules. The GC oven temperature was programmed as follows: firstly 70 °C for 2 min, then increased to 200 °C at a rate of 5 °C min^−1^ and finally maintained for 2 min. The temperature of the injector was adjusted to 230 °C, while the flow rate of the carrier helium gas was maintained at 1 mL min^−1^, and a split ratio of 10:1 was employed. The ion source and interface were maintained at temperatures of 200 °C and 240 °C, respectively. The solvent delay was configured to a duration of 1.5 min. The mass spectrometer was utilized in an electron impact mode with an energy of 70 eV, and mass spectra were obtained using a scan mode encompassing a range of 35–400 *m*/*z*. The volatile molecules were identified based on the data of the National Institute Standard and Technology Library (version 5).

#### 2.3.4. XRD Analysis

X-ray diffractograms of ICs were obtained via an XRD (D8 ADVANCE/TSM, Bruker Corporation, Billerica, MA, USA) using Cu-Kα radiation at an accelerated voltage of 40 kV and a current of 40 mA. The scans were conducted within the 2*θ* angle range of 2–30° with an incremental step of 0.02°.

#### 2.3.5. FT-IR Analysis

The FT-IR spectra were collected between 4000 and 400 cm^−1^ via an FT/IR-6800 (JASCO Corporation, Tokyo, Japan) at a resolution of 4 cm^−1^ for 64 consecutive scans. HEO was pasted on a potassium bromide plate and ICs were ground with KBr powders followed by a pressing into an approx. 1 mm thick pellet.

#### 2.3.6. Determination of Water Contact Angle

The hydrophilic property of the prepared ICs was determined by measuring the water contact angle using a sessile drop technique. The samples were prepared according to our previous study with few modifications [28]. Briefly, approx. 0.1 g ICs were placed evenly across the surface of a glass slide, followed by a press at 20 MPa for 30 s. Afterwards, the free ICs were gently purged with air via a miniature rubber blower. An automatic Contact Angle Meters DropMaster (DMs-401, Kyowa Interface Science Co., Ltd., Saitama, Japan) was used to measure the water contact angle. A total of 2 µL Milli-Q water was deposited on the surface and the contact angle was measured using a θ/2 method after equilibration.

#### 2.3.7. Anti-*S. aureus* Analysis

The anti-*S. aureus* activity of ICs was assessed via a colony counting method according to the methodology described by Cui et al. [25] with a few modifications. The cells of *S. aureus* were grown on a shaker (BR-23FH, TAITEC Corporation, Saitama, Japan) at 100 rpm min^−1^ and 30 °C for 24 h. The inoculum was diluted and injected into a glass tube containing 4 mL TSB to provide a final density of 1 × 10^6^ cell mL^−1^. Then, a certain weight of ICs (0.02, 0.04 and 0.08 g) was added to the glass tube (to achieve the doses of 5, 10 and 20 g L^−1^). Subsequently, the glass tube was shaken at 100 rpm min^−1^ and 30 °C for 24 h. Finally, 20 µL of inoculum diluted from the glass tube was spread on a TSA plate and the population of *S. aureus* was counted. As controls, glass tubes added with HEO, β-CD and 2-HP-β-CD were prepared, respectively. In addition, a glass tube merely containing TSB and *S. aureus* was set as a blank. The inhibitory rate was calculated by the following equation.

$$\mathrm{Inhibitory}\;\mathrm{rate}\;(\%)=\frac{\mathrm{Population}\;\mathrm{of}\;S.\mathrm{aureus}\mathrm{in}\mathrm{blank}\;–\mathrm{Population}\;\mathrm{of}\;S.\;\mathrm{aureus}\mathrm{in}\;\mathrm{sample}\;}{\mathrm{Population}\;\mathrm{of}\;S.\;\mathrm{aureus}\mathrm{in}\mathrm{blank}}\times \;100$$

#### 2.3.8. Statistical Analysis

The results obtained in this study were analyzed according to SPSS software (28.0 version, IBM Corporation, Armonk, NY, USA) with a one-way analysis of variance (ANOVA). The difference at *p* < 0.05 was considered significant via the least significant difference (LSD) test. The data were reported as means ± standard deviation. All the experiments were repeated three times.

## 3. Results and Discussion

### 3.1. Complexation Efficiency Analysis

**Figure 1:**
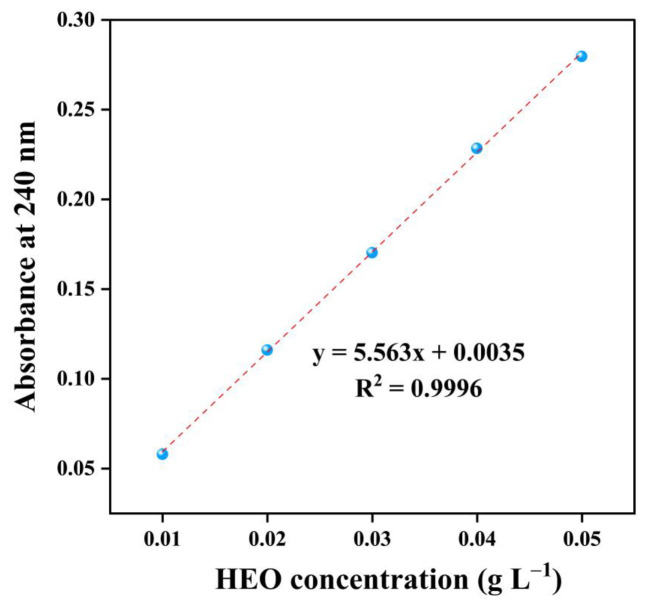
Calibration curve of HEO concentration versus absorbance at 240 nm.

Complexation of HEO into the interior cavities of β-CD and 2-HP-β-CD was conducted to overcome the disadvantages such as instability even in an ambient condition. In this study, RY, EF, and LC were applied to assess the complexation efficiency of the ultrasound-assisted kneading method. Figure 1 depicts the calibration curve used for the calculation of the weight of embedded HEO in ICs. Table 1 displays the RY, EF, and LC values of HEO/β-CD ICs, HEO/β-CD ICs control, HEO/2-HP-β-CD ICs, and HEO/2-HP-β-CD ICs control. In general, the RY value for HEO/β-CD ICs was extraordinarily high as compared to some recent other works. Hădărugă et al. [23] used β-CD to complex common nase oil via the traditional kneading method and reported RY values of 77.7%, which was substantially lower than this work. Ma et al. [29] conducted a Box–Behnken design on the β-CD and clove oil ICs via a co-precipitation method and found the highest yield of RY was as low as 75.5%. However, a relatively low RY value of HEO/2-HP-β-CD ICs was noticed in contrast with HEO/β-CD ICs and this difference was as well reflected in the EF value. The main reason for this phenomenon is the certain solubility of 2-HP-β-CD in ethanol, which led to a partial dissolution and loss of 2-HP-β-CD ICs during the washing process. LC is regarded as the prime criterion for estimating complexation efficiency as it more accurately reflects the HEO amount in the ICs which directly influences the magnitude of anti-*S. aureus* property [30]. Obviously, in contrast to samples prepared solely through a kneading method, the utilization of an ultrasound-assisted procedure yielded higher LC values. This indicates a greater entrapment of HEO molecules by the hollow cavities of host molecules. In the traditionally used kneading methods, an extended duration of operation is typically required to achieve a more thorough dispersion of HEO molecules [23,24]. This dispersion is necessary for the molecules to effectively penetrate the cavities of host molecules. However, this extended operation time would be a minor drawback in large-scale manufacturing due to the associated increase in time cost. This study introduces a novel three-stage complexation procedure with a total duration of only 15 min. The primary purpose of the initial 5 min kneading process was to achieve a relatively uniform dispersion of the HEO molecules. Subsequently, the 5 min ultrasonic operation further facilitated the dispersion of HEO molecules into smaller particles driven by high energy. This process also accelerated the entry of guest molecules into the cavities of host molecules. The final 5 min kneading process mainly aimed to ensure the full complexation of the remaining HEO molecules. This step was necessary since the high energy involved in the sonication phase may cause a partial release of HEO molecules that already entered the cavities of host molecules. The results of RY, EF, and LC values showed that the ultrasound-assisted kneading method had a superior complexation efficiency compared to the conventional kneading method. In addition, the LC of HEO/2-HP-β-CD ICs was higher than that of HEO/β-CD ICs, presumably due to the improved inclusion ability of 2-HP-β-CD [31,32,33]. As a result, the inhibitory rate of HEO/2-HP-β-CD ICs on *S. aureus* should be theoretically higher than that of HEO/β-CD ICs, as will be described later in the section on anti-*S. aureus* analysis.

### 3.2. SEM Analysis

**Figure 2:**
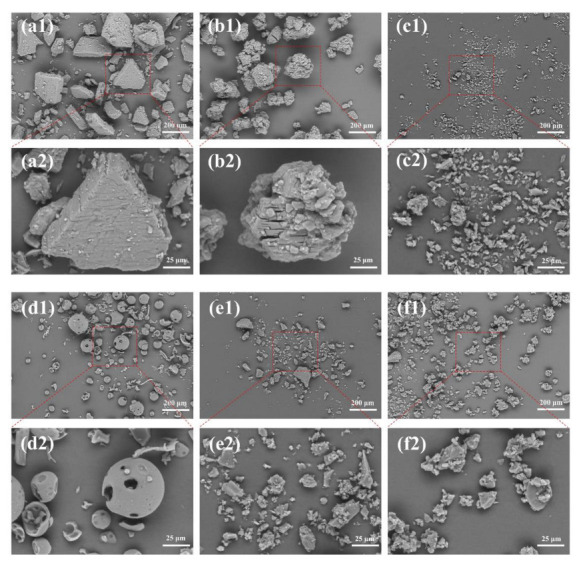
SEM images of β-CD ((**a1**) at 200 times magnification and (**a2**) at 800 times magnification, hereafter), β-CD blank (**b1**,**b2**), HEO/β-CD ICs (**c1**,**c2**), 2-HP-β-CD (**d1**,**d2**), 2-HP-β-CD blank (**e1**,**e2**) and HEO/2-HP-β-CD ICs (**f1**,**f2**).

Figure 2 shows the SEM images of morphological and dimensional structures of fabricated ICs. β-CD exhibited irregularly shaped masses or lamellar plates of different sizes. Notably, the surface displayed tiny particles, as well as fractures or striations. After complexation, the prepared HEO/β-CD ICs showed a dramatic change in appearance, presenting rhomboidal shapes with a significant decrease in size compared with the β-CD, as reported by Barbieri et al. [34]. Nevertheless, there were no observable fractures or striations, indicating that the HEO molecules were adequately protected as demonstrated by Anaya-Castro et al. [35]. The surface of β-CD blank exhibited a high degree of similarity to that of β-CD, indicating that the change in shape was caused solely by the kneading process as well observed in the study of Pinheiro et al. [36]. 2-HP-β-CD displayed numerous spherical or hemispherical structures with several holes or cavities, which were conducive to the embedding of HEO molecules. However, HEO/2-HP-β-CD ICs appeared as irregular flaky structures with small particles generated on the surface consistent with the result observed by Pinheiro et al. [36]. The morphology of the 2-HP-β-CD blank presented an irregular shape of varying sizes, probably caused by the rupture after receiving the mechanical kneading force applied to the original 2-HP-β-CD. Michalska et al. [37] and Xi et al. [38] explained that the formation of ICs would alter the original conformation of the host material, and the difference in microstructure could be used as a sign of the formation of ICs, suggesting the successful embedding of HEO in this study.

### 3.3. GC-MS Analysis

**Figure 3:**
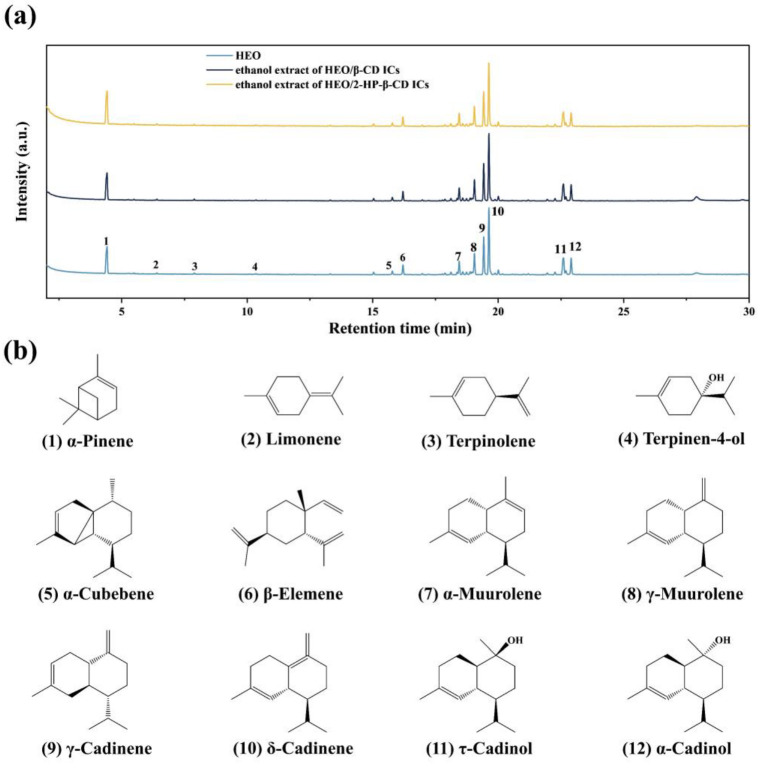
Total ion chromatograms of HEO, ethanol extracts of HEO/β-CD ICs and HEO/2-HP-β-CD ICs (**a**); twelve typical molecules labeled from “1” to “12” identified from HEO (**b**).

The HEO molecules embedded in ICs were identified by GC-MS analysis. The total ion chromatograms of HEO, ethanol extracts of HEO/β-CD ICs and HEO/2-HP-β-CD ICs are depicted in Figure 3a. The total ion chromatograms of ethanol extracts of HEO/β-CD ICs and HEO/2-HP-β-CD ICs exhibited a high degree of similarity to that of HEO. This observation suggests that the HEO molecules effectively penetrated the hydrophobic inner cavities of host molecules during the process of complexation. The volatile bioactive compounds were identified as the compounds discovered within the retention time range of 4 to 24 min, which matches the temperature of 80 to 180 °C of the GC oven. Figure 3b shows the structures of twelve typical volatile bioactive molecules labeled from “1” to “12” detected in the total ion chromatograms of HEO. It is reported that terpenes and terpenoids functioned as the primary bioactive molecules of EOs and have a significant potential for anticancer, antimicrobial, anti-inflammatory, antioxidant, antiallergic, and analgesic [39,40]. The molecules labeled from “1” to “3” are typical monoterpenes, whereas molecule “4” belongs to monoterpenoids containing oxygen that is constructed via biochemical modifications. Clearly, the intensity of the molecules labeled from “5” to “12” corresponding to sesquiterpenes and sesquiterpenoids, increases dramatically compared with the monoterpenes and monoterpenoids labeled from “1” to “4”. Particularly, it has been shown that sesquiterpenes and sesquiterpenoids were exceptionally promising for the growth suppression of *S aureus* [41,42] that aligns well with our expectation and may provide the prepared ICs with a satisfactory anti-*S aureus* result.

### 3.4. XRD Analysis

**Figure 4:**
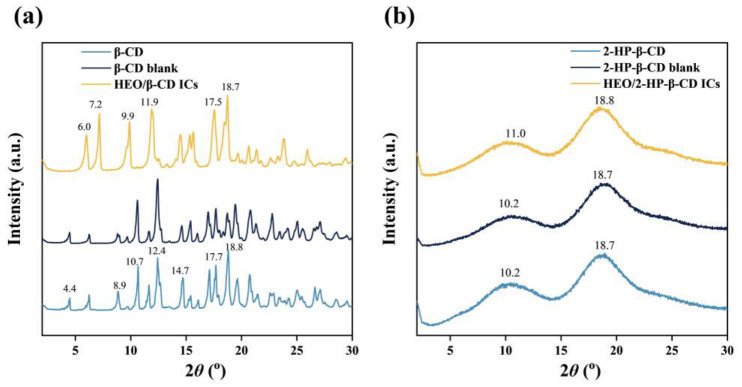
XRD patterns of β-CD, β-CD blank, HEO/β-CD ICs (**a**) and 2-HP-β-CD, 2-HP-β-CD blank and HEO/2-HP-β-CD ICs (**b**).

XRD is a useful technique for the detection of crystalline states. XRD patterns of β-CD, β-CD blank and HEO/β-CD ICs are presented in Figure 4a. Apparently, the XRD pattern of HEO/β-CD ICs exhibited notable differences compared to that of β-CD. However, a significantly high degree of similarity between the β-CD blank and β-CD was present, signifying the ultrasound-assisted kneading operation had no impact on the crystal structure as observed in the SEM images. The XRD pattern of the β-CD displayed five main peaks at 10.7°, 12.4°, 14.7°, 17.7° and 18.8°, whereas the HEO/β-CD ICs showed sharp peaks at 6.0°, 7.2°, 9.9°, 11.9°, 17.5° and 18.7° in assistance with our previous study [28] and in Pinheiro et al. [36]. Ogata et al. [43] provided an explanation that the significant fluctuations observed in these peaks can be attributed to the structural transition, which indicates the successful formation of ICs. As well, alterations such as the vanishing of the peaks at 4.4°, 8.9° and 10.7° and the emergence of a new peak at 7.2° suggested the changes in the crystal structure, as similarly stated by Abarca et al. [44]. Furthermore, the intensity of the peaks between the diffraction angle 2*θ* of 12° and 20° changed to some extent after complexation, and these represented the changes in crystallographic orientation resulting from the entrapment of HEO molecules by β-CD cavities. As illustrated in Figure 4b, the diffraction patterns of 2-HP-β-CD and 2-HP-β-CD blank were characterized by two broad dispersion peaks at 10.2° and 18.7° related to the non-crystalline form, in line with observations of Sun et al. [16]. The XRD pattern of the HEO/2-HP-β-CD ICs was almost completely superimposed on that of 2-HP-β-CD; however, a slight shift of the two broad picks with a reduced intensity was noticed and Michalska et al. [37] explained this shift implied an interaction between guest molecules and 2-HP-β-CD, resulting in the formation of the ICs.

### 3.5. FT-IR Analysis

**Figure 5:**
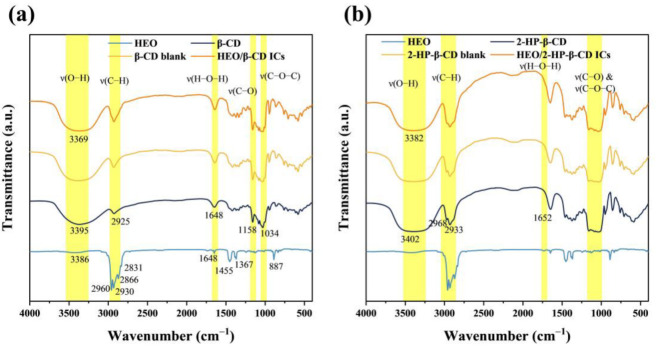
FT-IR spectra of HEO, β-CD, β-CD blank, HEO/β-CD ICs (**a**) and 2-HP-β-CD, 2-HP-β-CD blank and HEO/2-HP-β-CD ICs (**b**).

FT-IR was used to demonstrate the interaction between host materials and HEO molecules in the process of ICs formation in terms of peak shape, position and intensity. Figure 5a shows the FT-IR spectra of HEO, β-CD, β-CD blank and HEO/β-CD ICs. HEO displayed strong four absorption peaks from 2960 to 2831 cm^−1^, corresponding to the asymmetrical stretching vibration of –CH_3_, symmetrical stretching vibration of –CH_3_, asymmetrical stretching vibration of –CH_2_– and symmetrical stretching vibration of –CH_2_–, respectively. The broad weak absorption peak at 3386 cm^−1^ was for O–H stretching vibrations and in line with the findings obtained from the GC-MS detection where the molecules “4”, “11” and “12” containing O–H merely accounted for a minority of the detected molecules. The weak absorption peaks observed approx. A total of 1648 cm^–1^ were exhibited by the C=C stretching vibrations. The mild peak located at 1455 and 1367 cm^–1^ arose from the –CH_2_– and –CH_3_ scissoring vibrations. The low but sharp peak at 887 cm^–1^ was likely associated with –CH_2_– bending vibrations of the vinylidene structure from the terpenes and terpenoids. The absorption bands of β-CD were observed at 3395, 2925, 1648, 1158 and 1034 cm^−1^, corresponding to the vibration of symmetrical and asymmetrical stretching of the O–H, C–H stretching vibrations, H–O–H bending, C–O stretching vibration and symmetric stretching of glycosidic C–O–C, respectively [44]. Obviously, the FT-IR spectra of β-CD blank as well as HEO/β-CD ICs revealed a high degree of resemblance to that of β-CD, except for the appearance of a new weak pick located at 2960 cm^−1^ (asymmetrical stretching vibration of –CH_3_) generated by the embedded HEO molecules. Furthermore, the O–H peak position of HEO/β-CD ICs shifted to a lower wavenumber compared to the β-CD, explained by Kayaci and Uyar [45] that this phenomenon denoted the occurrence of hydrogen bonding interactions between guest and host molecules, suggesting the successful formation of ICs. Figure 5b illustrates the FT-IR spectra of HEO, 2-HP-β-CD, 2-HP-β-CD blank and HEO/2-HP-β-CD ICs. Noticeably, the spectra of 2-HP-β-CD were nearly identical to that of β-CD due to their structural similarity. However, the C–O and C–O–C peaks of 2-HP-β-CD almost overlap owing to the substitution of hydrogen with hydroxypropyl. Likewise, the spectra of HEO/2-HP-β-CD ICs were predominantly characterized by the bands originating from 2-HP-β-CD and the position of the O–H absorption peak shifted towards a lower wavenumber after complexation. Additionally, as a result of a higher water solubility and having –OH groups affixed to more flexible alkyl moieties, the 2-HP-β-CD broad band corresponding to the stretching vibration of O–H was more intense than that of β-CD [23]. Moreover, compared with HEO/β-CD ICs, the increased intensity for the broad O–H peak of HEO/2-HP-β-CD ICs was likely caused by a better complexation ability of 2-HP-β-CD for guest molecules as reported by Nuchuchua et al. [46] who found the 2-HP-β-CD had a stronger entrapping capacity than other cyclodextrins, agreeing with the LC result in the part of complexation analysis.

### 3.6. Water Contact Angle Analysis

**Figure 6:**
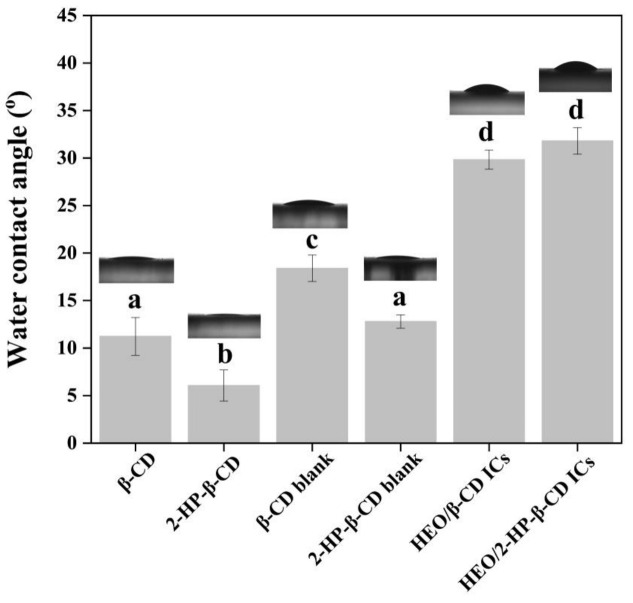
Water contact angles of β-CD, HEO/β-CD, β-CD blank, 2-HP-β-CD blank, HEO/β-CD ICs and HEO/2-HP-β-CD ICs. Different superscripts indicate significant differences (*p* < 0.05).

The complexation of HEO with β-CD and 2-HP-β-CD serves to mitigate its inherent instability and could facilitate the integration with other hydrophilic biodegradable materials for a broad spectrum of potential applications. The hydrophilicity of the ICs was determined by conducting water contact angle measurements, and the corresponding results are presented in Figure 6. The contact angle of β-CD was 11.2°, while a smaller value 6.1° of 2-HP-β-CD was exhibited due to its higher water affinity. However, a minor increase in contact angles was reflected for both blank samples, presumably due to the changes in surface morphology after a kneading process. The lamellar β-CD and spherical 2-HP-β-CD are convinced to display a certain amount of microscopic roughness when subjected to pressure on a glass slide where they were placed. However, the blank samples underwent a kneading process, resulting in the formation of aggregates or smaller crushed shapes. Consequently, when they were exposed to pressure on the glass slide, their structure became microscopically denser, and this led to a minor increase in the subsequent water contact angle measurements. The contact angles of HEO/β-CD ICs and HEO/2-HP-β-CD ICs were calculated to be 29.8° and 31.8°, respectively. Notably, after the complexation, the contact angles of prepared ICs exhibited a discernible rise, which could be ascribed to the presence of hydrophobic HEO molecules within the ICs. Nonetheless, they were considerably below 90°, revealing a good hydrophilicity of the prepared ICs. In addition, a higher contact angle of HEO/2-HP-β-CD ICs was displayed with a comparison to the HEO/β-CD ICs, this could be supported by a higher LC value for HEO/2-HP-β-CD ICs since it represents a higher proportion of embedded HEO molecules.

### 3.7. Anti-S. aureus Analysis

To investigate the anti-*S. aureus* activity of the fabricated ICs, a colony counting method was performed and the results are present in Table 2. The results clearly indicate that HEO exhibited a substantial inhibitory effect against *S. aureus*, even at a dose of 5 g L^−1^ in the TSB medium. The observed low inhibitory rate of β-CD and 2-HP-β-CD suggests that these host molecules may impede the uptake of nutrients by *S. aureus*, leading to a modestly reduced growth rate compared to the blank sample. This effect was more pronounced for β-CD, perhaps due to its limited solubility in water. However, due to the fact that they are oligosaccharides, it is impossible to fundamentally inhibit the growth of *S. aureus*. In contrast, the addition of ICs significantly slowed the proliferation of *S. aureus*, particularly the HEO/2-HP-β-CD ICs, which corresponded with the higher LC value. When the dose was 20 g L^−1^, the inhibitory rate was as high as 100%. In addition, the performance of anti-*S. aureus* activity indirectly revealed the HEO molecules were effectively embedded within the hollow cavities of β-CD and 2-HP-β-CD. As both the guest and host materials are classified as GRAS, ICs fabricated by the ultrasound-assisted kneading method in this study are believed to provide a promising alternative for the use of green anti-*S. aureus* agents in the food packaging industry.

## 4. Conclusions

This study developed green anti-*S. aureus* ICs containing HEO using β-CD and 2-HP-β-CD as host materials. The ultrasound-assisted kneading method was successfully applied for the complexation of HEO and a higher complexation efficiency was achieved with a comparison to the commonly used kneading method. The results of SEM, GC-MS, XRD and FT-IR suggested the HEO molecules were well entered into the cavities of host materials for the formation of ICs. As well, a significant anti-*S. aureus* activity of these ICs was exhibited, especially the HEO/2-HP-β-CD ICs due to a higher LC value. Importantly, inhibitory rates of 99.8% for HEO/β-CD ICs and 100% for HEO/2-HP-β-CD ICs were achieved when the dose in the TSB medium was 20 g L^−1^. In addition, the measurement of water contact angles validated the hydrophilicity of the produced ICs. This characteristic would enhance their ability to blend with hydrophilic biodegradable substances for diverse food packaging utilizations. Additional research should be conducted in the future to investigate the release characteristics of HEO molecules from ICs under different environmental conditions, such as variations in humidity and temperature.

## Figures and Tables

**Table 1:** Complexation efficiency of HEO/β-CD ICs, HEO/β-CD ICs control, HEO/2-HP-β-CD ICs, and HEO/2-HP-β-CD ICs control.

	RY (%)	EF (%)	LC (%)
HEO/β-CD ICs	92.5 ± 1.0 ^a^	78.0 ± 2.7 ^a^	11.9 ± 0.5 ^a^
HEO/β-CD ICs control	90.1 ± 1.7 ^b^	52.3 ± 2.0 ^b^	8.2 ± 0.4 ^b^
HEO/2-HP-β-CD ICs	80.8 ± 1.2 ^c^	73.7 ± 3.2 ^a^	12.9 ± 0.4 ^c^
HEO/2-HP-β-CD ICs control	78.9 ± 0.6 ^c^	58.9 ± 1.7 ^c^	10.6 ± 0.3 ^d^

Different superscripts within a column indicate significant differences (*p* < 0.05).

**Table 2:** Inhibitory rate of HEO, β-CD, 2-HP-β-CD, HEO/β-CD ICs and HEO/2-HP-β-CD ICs against *S. aureus* at doses of 5, 10 and 20 g L^−1^.

	Inhibitory Rate (%)		
	5 g L^−1^	10 g L^−1^	20 g L^−1^
HEO	100.0 ± 0.0 ^a^	100.0 ± 0.0 ^a^	100.0 ± 0.0 ^a^
β-CD	16.2 ± 0.6 ^b^	18.2 ± 1.5 ^b^	20.9 ± 1.2 ^b^
2-HP-β-CD	6.0 ± 5.9 ^c^	12.1 ± 1.9 ^c^	17.9 ± 1.3 ^c^
HEO/β-CD ICs	58.0 ± 3.1 ^d^	86.5 ± 1.1 ^d^	99.8 ± 0.2 ^a^
HEO/2-HP-β-CD ICs	72.8 ± 3.6 ^e^	93.0 ± 0.8 ^e^	100.0 ± 0.0 ^a^

Different superscripts within a column indicate significant differences (*p* < 0.05).

## Data Availability

The datasets generated for this study are available on request to the corresponding author.
